# NOX2β: A Novel Splice Variant of NOX2 That Regulates NADPH Oxidase Activity in Macrophages

**DOI:** 10.1371/journal.pone.0048326

**Published:** 2012-10-31

**Authors:** Craig B. Harrison, Stavros Selemidis, Elizabeth Guida, Paul T. King, Christopher G. Sobey, Grant R. Drummond

**Affiliations:** 1 Vascular Biology and Immunopharmacology Group, Department of Pharmacology, Monash University, Clayton, Victoria, Australia; 2 Department of Medicine/Respiratory Medicine, Monash Medical Centre, Clayton, Victoria, Australia; Julius-Maximilians-Universität Würzburg, Germany

## Abstract

Nox2 oxidase is one isoform in a family of seven NADPH oxidases that generate reactive oxygen species (ROS) and thereby contribute to physiological and pathological processes including host defense, redox signaling and oxidative tissue damage. While alternative mRNA splicing has been shown to influence the activity of several Nox-family proteins, functionally relevant splice variants of Nox2 have not previously been identified. We immunoscreened several mouse tissues and cells for the presence of truncated Nox2 proteins and identified a 30 kDa protein in lung, spleen and macrophages. RT-PCR analysis of mRNA from primary and immortalised (RAW264.7) mouse macrophages, and from human alveolar macrophages, identified a truncated Nox2 transcript which, upon sequence analysis, was found to be a product of the ‘exon skipping’ mode of alternative splicing, lacking exons 4–10 of the Nox2 gene. The predicted protein is comparable in size to that identified by immunoscreening and contains two transmembrane helices and an extended cytosolic C-terminus with binding sites for NADPH and the Nox organiser protein p47phox. Importantly, selective siRNA-mediated knockdown of the transcript reduced expression of the 30 kDa protein in macrophages, and suppressed phorbol ester-stimulated ROS production by 50%. We thus provide the first evidence that Nox2 undergoes alternative mRNA splicing to yield a 30 kDa protein – herein termed Nox2β – that regulates NADPH oxidase activity in macrophages from mice and humans. The discovery of Nox2β paves the way for future examination of its role in physiological and pathological processes.

## Introduction

The Nox2 isoform of the NADPH oxidase family of superoxide generating enzymes is expressed in many cell types and participates in a wide range of cellular processes including pathogen clearance [1], signal transduction [2], [3], apoptosis [4], proliferation [5], [6] and oxygen sensing [7]. Heightened activity of Nox2 oxidase, leading to oxidative stress and tissue damage, has been described under various pathophysiological conditions including those associated with acute infections by micro-organisms [8], [9] and in chronic diseases such as hypertension [10], atherosclerosis [11], ischemia-reperfusion injury [12], [13] and cancer [14]. Conversely, a reduction in Nox2 oxidase activity due to mutations in the genes that encode Nox2 (Cybb), or any one of the three NADPH oxidase regulatory subunits, p22phox, p47phox or p67phox, underpins the condition known as chronic granulomatous disease (CGD), characterised by an inability of phagocytes to mount a respiratory burst to kill ingested pathogens, and leading to severe life-threatening infections by opportunistic bacteria and fungi [15], [16].

Recent studies demonstrate that several members of the Nox gene family including Nox1 [17], [18], Nox4 [19] and Nox5 [20], are subject to varying degrees of alternative mRNA splicing. Furthermore, for all of these genes it has been shown that one or more of the alternative splice variants are translated into protein and play functional roles in either suppressing superoxide generation (i.e. by acting as dominant-negatives) or in generating reactive oxygen species (ROS) by acting as oxidases in their own right. By contrast, only one splice variant has been described for Nox2 (denoted Nox2S [21]), but there is currently no evidence that this variant is translated into protein.

In the present study we immunoscreened a series of mouse tissues for the presence of truncated Nox2 proteins, and then further examined those tissues for evidence of alternative Nox2 mRNA splicing. We discovered a 30 kDa Nox2 protein fragment in lung and spleen, and then identified macrophages to be the likely cellular source of the protein. RT-PCR also revealed that macrophages express a Nox2 mRNA fragment that lacks exons 4 to 10 of the full length Nox2 gene. The predicted product of this truncated mRNA sequence is compatible in size to the protein fragment observed in the same cells, and consists of a short intracellular N-terminus, two hydrophobic domains that are predicted to be membrane spanning, and an extended cytosolic C-terminal tail that contains binding sites for the electron donor, NADPH, as well as docking regions for the NADPH oxidase ‘organiser’ protein, p47phox. Importantly, selective siRNA-mediated knockdown of the truncated Nox2 sequence markedly reduced ROS production in macrophages, suggesting that the protein plays a key role in supporting NADPH oxidase activity in these cells. As only the second splice variant of Nox2 identified to date, we propose that it be named Nox2β.

## Materials and Methods

### Tissue Isolation and Cell Culture

Tissues and primary cells were isolated from age-matched (10–12 weeks) male C57Bl/6J (wild type) and Nox2^−/y^ mice or from age-matched (19–26 weeks) male wild type, ApoE^−/−^ and Nox2^−/y^/ApoE^−/−^ mice. Nox2^−/y^ mice were originally generated by Prof. Mary Dinauer [22] and were backcrossed with ApoE^−/−^ to generate double knockout Nox2^−/y^/ApoE^−/−^ mice [11]. All mice were maintained on tap water and normal chow except for the 19–26 week old C57Bl/6J, ApoE^(−/−)^ and Nox2^(−/y)^ApoE^(−/−)^ mice, which at 5 weeks of age, received either 14 or 21 weeks of high-fat (21%) diet (HFD). Mice were housed in either standard cages or sterile microisolator cages under a 12 h light–dark cycle, kept at 20±2°C. All animal procedures were conducted with the approval of the Monash University School of Biomedical Sciences Animal Ethics Committee.

**Figure 1:**
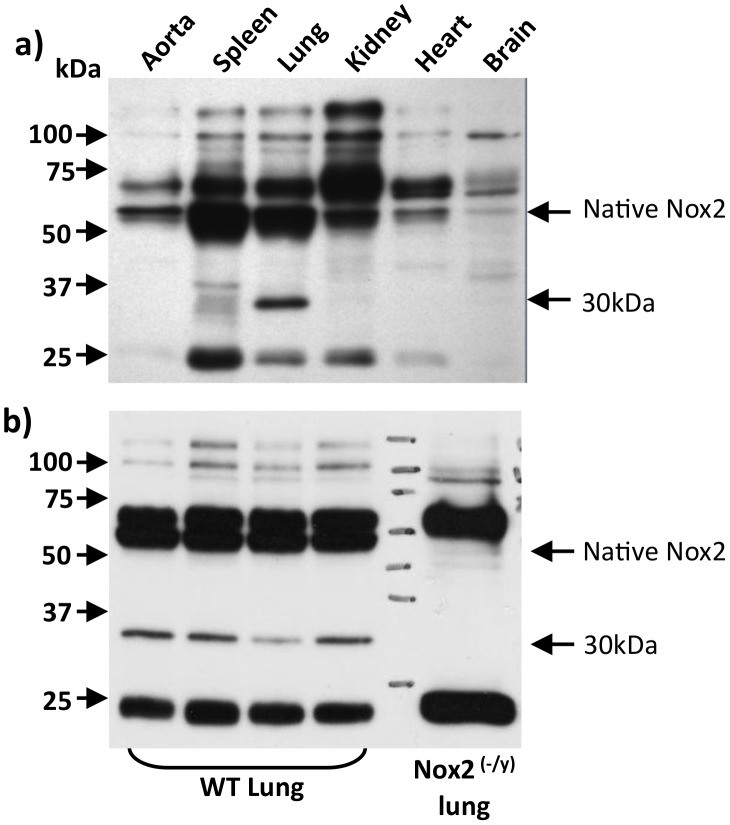
Evidence for a truncated Nox2 protein (Nox2β) in mouse tissues. Western blot images showing protein expression of native Nox2 and the novel ∼30 kDa protein, Nox2β, in (a) various tissues and organs taken from wild type mice and (b) lung tissue taken from wild type and Nox2^−/y^ mice. Note, in (b) the truncated ∼30 kDa band is absent in tissue samples from Nox2^−/y^ mice. For data shown in (b), lung samples were taken from 4 separate wild type mice.

**Figure 2:**
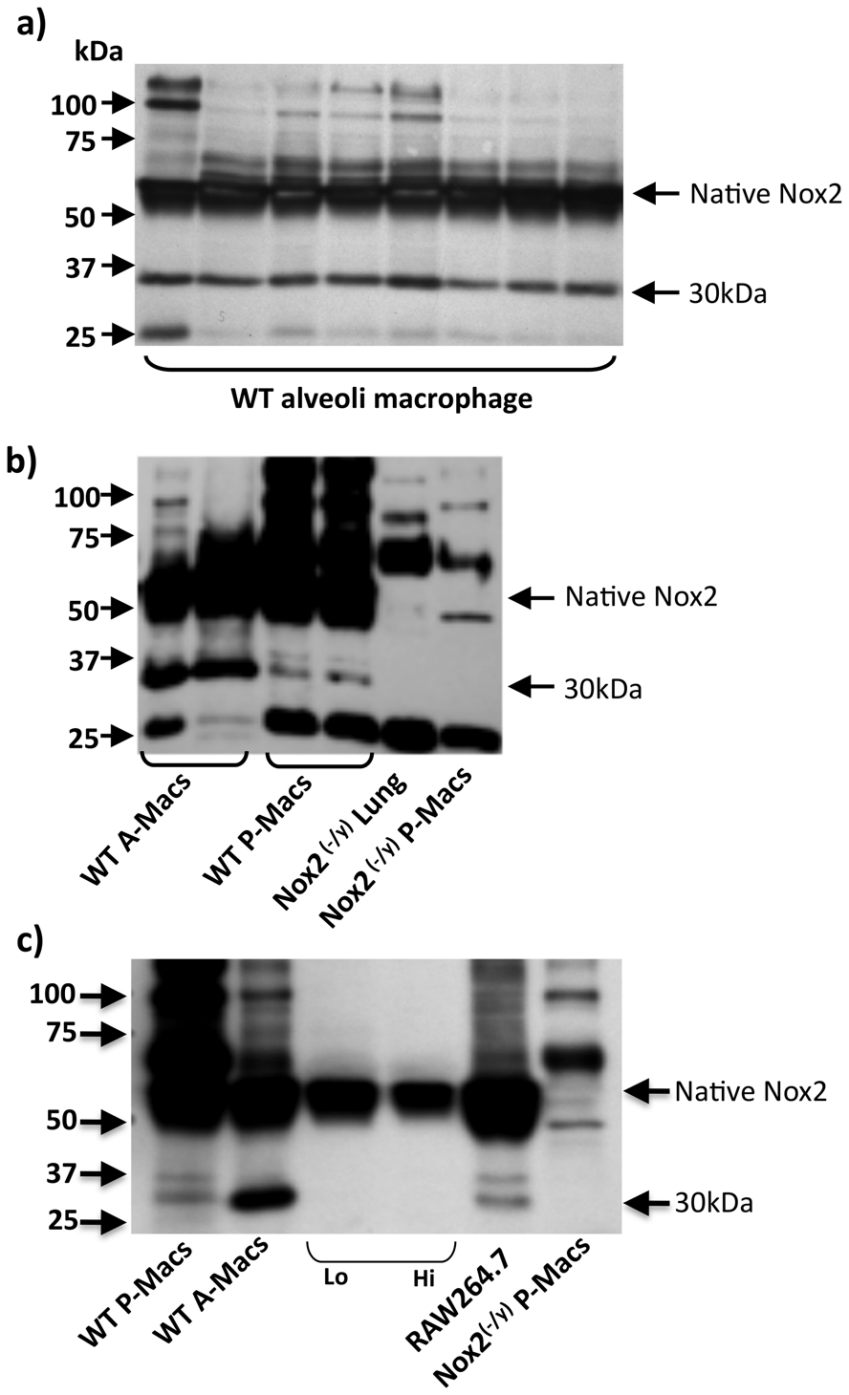
Evidence for a truncated Nox2 protein in macrophages. Western blot images showing protein expression of the novel 30 kDa variant of Nox2 and native Nox2 in: (a) alveolar macrophages from 8 individual wild type mice; (b) peritoneal macrophages, and for comparison, alveolar macrophages from 2 individual wild type mice and (c) Ly6C^lo^ and Ly6C^hi^ expressing monocyte subsets and the macrophage cell line RAW264.7. Note in (b) and (c) the lack of expression of the novel Nox2 variant in lung and peritoneal macrophages from Nox2^−/y^ mice.

Mice from which organs were removed, were firstly heparinized (100 µL, i.p.; David Bull Laboratories, Melbourne, Victoria, Australia) 20 min before they were anaesthetized with isoflurane (Baxtor Healthcare, Australia). Lungs and whole aorta were then removed, placed in 4°C Krebs-HEPES buffer (composition (in mmol/L): NaCl 118; KCl 4.7; KH_2_PO_4_ 1.2; MgSO_4_·7H_2_O 1.2; CaCl_2_ 2.5; NaHCO_3_ 25; glucose 11.7; HEPES 20, pH 7.4), cleared of surrounding fat and connective tissue and snap-frozen in liquid nitrogen for Western blotting.

**Figure 3:**
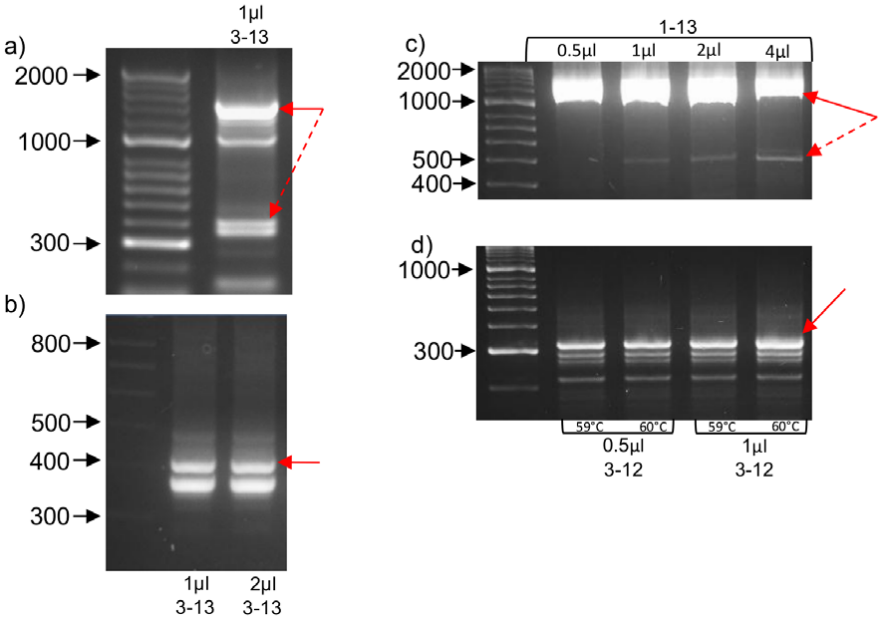
Expression of truncated transcripts of Nox2 in macrophages using RT-PCR and macrophage cDNA templates. (a) 1 µl of primary peritoneal macrophage cDNA template was amplified using a primer set including exon 3 forward and exon 13 reverse. (B) Re-amplification and nested PCR reactions were run using either 1 µl or 2 µl of cDNA isolated from the ∼400 base pair bands observed in (a) and with primer sets containing exon 3 forward and exon 13 reverse. (c) 0.5–4.0 µl of RAW264.7 cDNA was amplified using a primer set including exon 1 forward and exon 13 reverse. (D) Nested PCR reactions using either 0.5 or 1 µl cDNA eluted from the 500 bp band observed in (c) and with primer sets including an exon 3 forward and exon 13 reverse. Solid red arrows indicate bands, which were eluted and sequenced while broken arrows indicate eluted bands for nested PCR reactions. Note all samples were separated by running on 3% agarose gels.

Mice from which monocytes were isolated were overdosed with isoflurane and had their blood collected from the inferior vena cava. Monocytes were purified from whole blood using the EasySep® mouse monocyte enrichment kit (STEMCELL Technologies, Australia) according to the manufacturer’s protocol. Monocyte subsets (Ly6C^hi^ and Ly6C^lo^) were isolated via fluorescence-activated cell sorting flow cytometry.

Mice used for macrophage isolation were overdosed with isoflurane and then placed on their backs with their feet taped to a dissecting mat. For the isolation of alveolar macrophages, a thin shallow incision from the lower jaw to the top of the rib cage was made and the larynx was separated to expose the top of the trachea. The layer of smooth muscle covering the trachea was removed, a small incision made and a sheathed 21-gauge needle was inserted into the lumen. The lungs were repeatedly (5 times) lavaged with 300–400 µl of 37°C Krebs-HEPES buffer. The retrieved buffer was then incubated in a 5 ml cell culture flask for 3 hrs at 37°C for macrophage enrichment. Following the incubation period, the buffer was tipped out of the flask and the macrophages that remained adhered to the flask were washed with fresh Krebs-HEPES. Cells were then scraped in either 500 µl of Laemmli buffer (see Western blotting methods for recipe) and snap frozen in liquid nitrogen for Western blotting, or in 1 ml of fresh Krebs-HEPES and spun down to a palette (300 g for 5 min) for RNA extraction.

**Figure 4:**
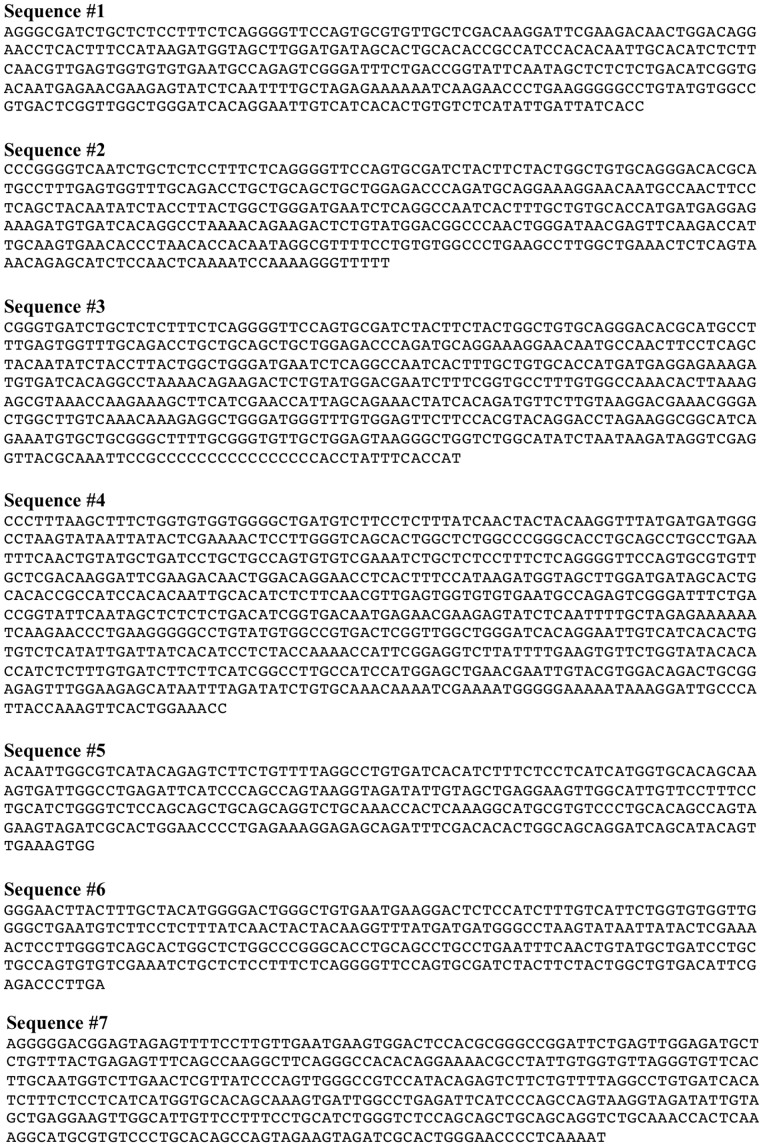
Sequencing data following RT-PCR.

For the isolation of peritoneal macrophages, a long, shallow incision was made from the bottom of the ribcage to the testes, removing the skin and hair but not breaking the abdominal wall. A small (∼4 mm in length) incision was made into the abdominal cavity, through which the tip of a 5 ml syringe was inserted. 4 ml of 37°C Krebs-HEPES buffer was injected into the peritoneum, which was clamped closed with forceps. The mouse was gently rolled from side to side, agitating the buffer within the cavity. The same syringe was then used to recover as much of the buffer as possible without damaging any internal organs. This process was repeated 5 times with fresh Krebs-HEPES buffer each time. The recovered buffer was then treated as outlined for the alveolar macrophages above.

A mouse macrophage cell line, RAW264.7 (Cat. No. TIB-71TM; ATCC, USA) was grown in Dulbecco’s Modified Eagle’s Medium (DMEM) containing 4.5 g/L D-glucose and 10% foetal calf serum (FCS). Cells were grown to 90% confluency, washed in warm (37°C) phosphate buffered saline (PBS), and then counted using the Countess® automated cell counter (Invitrogen) and homogenised as per the primary cells.

### Human Alveolar Macrophages

Human alveolar macrophages were obtained from subjects undergoing a bronchoscopy at Monash Medical Centre to investigate underlying lung disease with approval from the ethics committee of Southern Health/Monash Medical Centre. Written consent was obtained from all subjects. The bronchoscope was wedged in the right middle lobe and 25–50 ml of saline was washed into the airway then aspirated. Cells were washed twice with PBS before being suspended in culture medium (RPMI with 10% fetal calf serum with 100 units/ml penicillin and 100 micrograms/ml streptomycin) for ∼24 hr before use.

**Figure 5:**
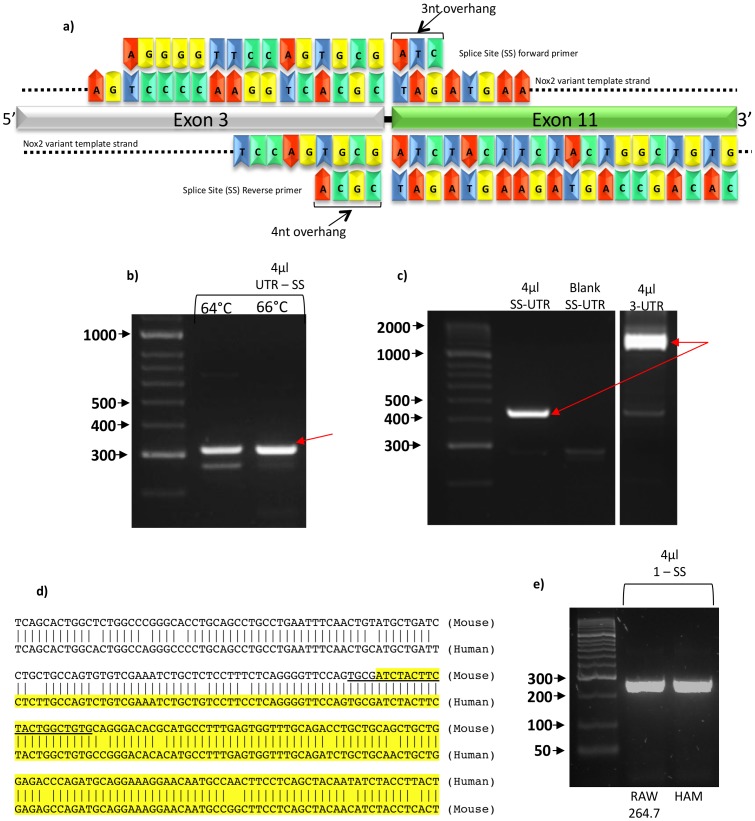
Splice variant specific primers. (a) Sequence and predicted binding sites of two splice site (SS) primers in the forward and reverse positions, designed to compliment the nucleotide sequence across the splice site between exon 3 and exon 11 of the splice variant sequence. Both primers were designed with small 3′ overhangs to prevent non-specific binding of the primers to native Nox2 cDNA. (b) Products of RT-PCR reactions using 1 µl of RAW264.7 cell cDNA template and primer sets containing either 5′ UTR or exon 1 forward and the reverse SS primer at varying annealing temperatures. (c) Products of RT-PCR reactions using 1 µl of RAW264.7 cell cDNA template and primer sets containing either the SS forward primer or a forward primer for exon 3 and a reverse primer for the 3′ UTR of Nox2 cDNA. Red arrows indicate DNA bands which were isolated for sequencing. (d) Alignment of the mouse and human Nox2 sequences revealing an extremely high degree of homology at the respective splice site junctions predicted to be formed by the deletion of exons 4–10. Shown in unhighlighted text is the nucleotide sequence for exon 3 and in yellow highlighted text is the sequence for exon 11. The underlined sequence shows the sequence of the splice site-specific reverse primer. (e) Products of RT-PCR reactions using 4 µl of either mouse RAW264.7 cDNA or human alveolar macrophage cDNA as templates and primer sets containing either the SS reverse primer and a forward primer for exon 1.

### RNA Extraction and Reverse Transcription

Peritoneal macrophages (from wild type and Nox2^−/y^ mice) and RAW264.7 cells were either lysed directly in culture flasks or from frozen pellets, and RNA was purified using the RNeazy Micro kit (Qiagen). RNA was eluted in RNase/DNase free water. RNA concentrations were measured using the NanoDrop spectrophotometer (Thermo Scientific) from 2 µl of sample. Reverse transcription (RT) was performed with 1 µg of RNA using Superscript III Reverse Transcription kit (Invitrogen). RT reactions were primed with Oligo (dT) (Invitrogen).

**Figure 6:**
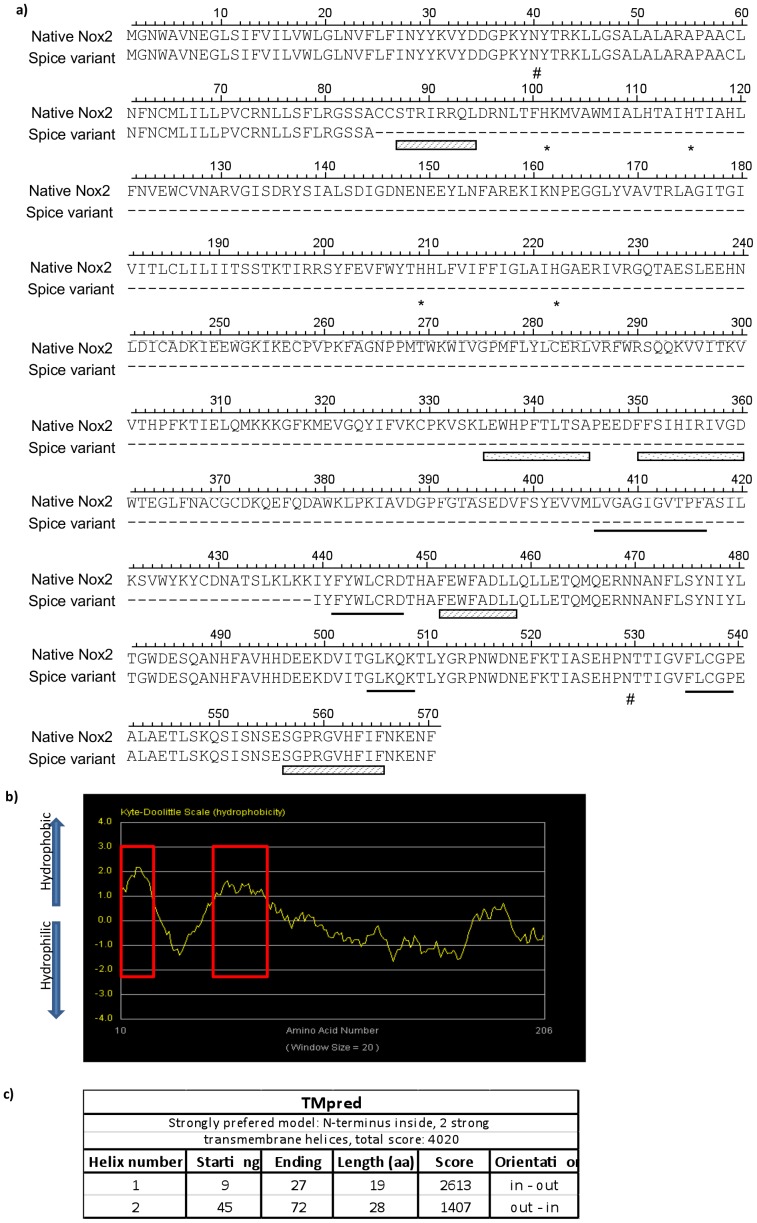
Nox2β and native Nox2 protein sequence alignment and bioinformatics. (a) The predicted amino acid sequence of the splice variant protein is aligned with the amino acid sequence of native Nox2 (Acc: BAE31076). Hyphens indicate region of spliced sequence. Asterisks indicate histidine residues reported to be important in heme binding. Underlined amino acids specify putative NADPH binding sites and hatched boxes signify predicted p47phox binding regions. Spotted boxes indicate the putative FAD binding motifs and the hash indicate predicted glycosylation sites. Alignment was achieved using DNASIS MAX software. (b) Hydrophobicity plot generated by “***The Molecular Toolkit:*** *Colorado State University”.* Red boxes indicate two regions of significant hydrophobicity.

### PCR

PCR was performed using GoTaq green master mix (Promega). Varying amounts of the cDNA templates (1–2 µl) were used in each reaction with 1 µM of each primer. PCR conditions were as follows (unless otherwise stated in the results section): 95°C for 5 min; 40 cycles of 95°C for 2 min, 59–66°C for 30 s and 72°C for 2 min; and 72°C for 5 min. Primers were designed against several regions of native Nox2 mRNA (GeneBank Accession number: NM_007807.4) using Invitrogen OligoPerfect™ Designer and are listed (5′ to 3′) below:

Exon 1 forward: AAC TGG GCT GTG AAT GAA GG.

Exon 3 forward: CAA CTG TAT GCT GAT CCT GCT G.

Exon 12 reverse: CGT CCA TAC AGA GTC TTC.

Exon 13 reverse: GGA TTC TGA GTT GGA GAT GCT C.

5′UTR forward: AGC ACA GTA GAG AAG AGA GGC A.

3′UTR reverse: CAT TTG GCA GCA TAC ACT GG.

Splice site (SS) forward: CCT CAC TTT CCAT ATC CGT ATT.

Splice site (SS) reverse: CAC AGC CAG TAG AAG TAG ATC GCA.

PCR products were loaded along with 7 µl of a DNA ladder (HyperLadder II, Bioline) onto agarose gels (2–4%) containing 0.1% SYBR safe DNA stain (Invitrogen). Bands of interest were isolated from the gels and DNA was extracted using either a Gel Extraction kit (Invitrogen) or the MicroElute Gel Extraction kit (Qiagen). DNA concentrations were measured using the NanoDrop spectrophotometer (Thermo Scientific) from 2 µl of sample. DNA was then either directly sequenced or used in further nested PCR reactions to increase yield and help clear the template of contaminating DNA which may have been amplified in the first PCR.

**Figure 7:**
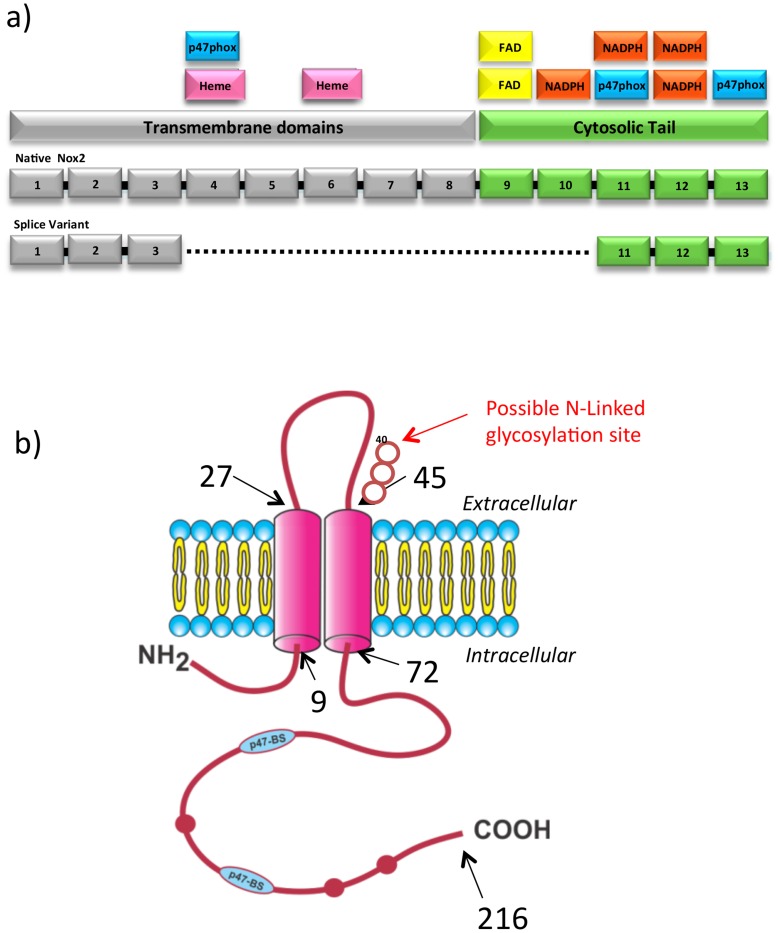
Predicted functional domains of Nox2β. (a) Schematic diagram of aligned Nox2β and native Nox2 proteins showing the locations of putative functional domains. Colour coding represents the transmembrane domains and the cytosolic tail. (B) Schematic diagram of Nox2β modelled using the TMpred software from European Molecular Biology Network (EMBnet). Black arrows and numbers indicate residue number at distinct points in the model. Red bulges in the protein chain represent conserved NADPH binding sites. Blue p47-BS show the approximate positioning of conserved p47phox binding sites. Red arrow indicated a potential N-linked glycosylation site at amino acid 40. The N-Y-T identifies the consensus sequence. (K. Hoffman and W. Stoffel (1993) TMbase - A database of membrane spanning proteins segments Biol. Chem. Hoppe-Seyler 374,166).

### Sanger Sequencing

DNA sequencing was performed using the PRISM® BigDye® Terminator v3.0 Cycle Sequencing Kit and a 3730 Genetic Analyser (both from Applied Biosystems). Sequencing reactions consisted of 10 ng of DNA template, 3.2 pmol of a sequencing primer, 1x sequencing buffer and 1x terminator mix made up to volume with deionized water. The cycle parameters were: 95°C for 5 min; 50 cycles of 95°C for 30s, 55°C for 10 s and 60°C for 5 min.

Sequencing primers are listed (5′ to 3′) below:

Exon 1 forward: AAC TGG GCT GTG AAT GAA GG.

Exon 3 forward: CAACTGTATGCTGATCCTGCTG.

Exon 12 reverse: CGT CCA TAC AGA GTC TTC.

Exon 13 reverse: GGA TTC TGA GTT GGA GAT GCT C.

5′UTR forward: AGC ACA GTA GAG AAG AGA GGC A.

3′UTR reverse: CAT TTG GCA GCA TAC ACT GG.

Splice site (SS) forward: CCT CAC TTT CCAT ATC CGT ATT.

Splice site (SS) reverse: CAC AGC CAG TAG AAG TAG ATC GCA.

Twenty µl of each reaction were injected into the DNA analyser fitted with a 50 cm capillary array and run for 28 min at 8.5 kV at 60°C. The electrokinetic conditions used were 15 s injection time and 25 volts/cm (total EKI - 375 V-s/cm). Electropherograms were viewed and interpreted using Sequence Scanner Software v1.0 (Applied Biosystems).

**Figure 8:**
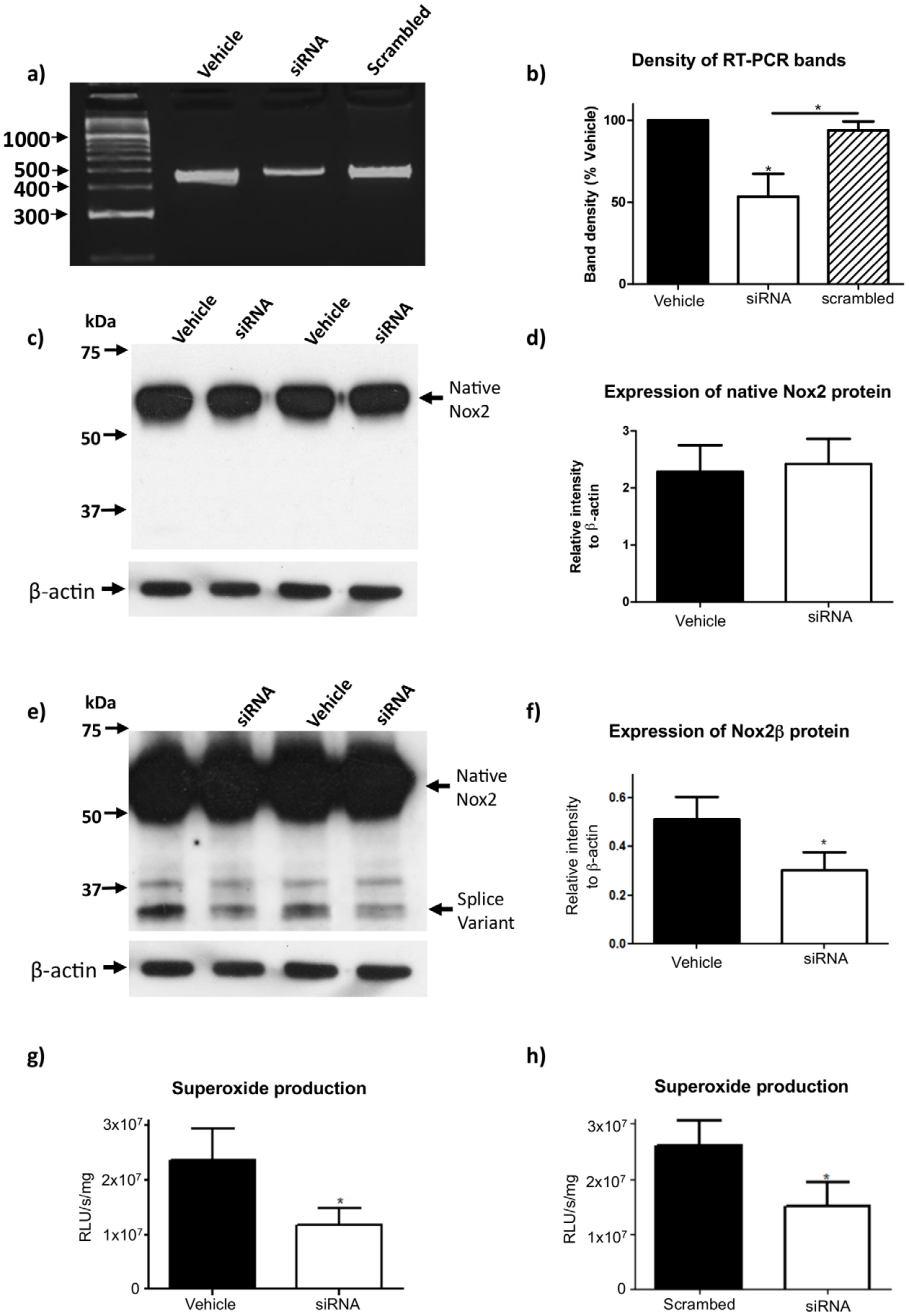
Superoxide generation by Nox2β. (a) Representative RT-PCR reaction employing the splice variant specific primers (SS - 3′UTR) and 3 µl RAW264.7 cell cDNA templates following either vehicle, siRNA or scrambled siRNA treatments and (b) the corresponding histogram of densitometric analysis of cDNA bands (n = 3). Representative Western blot images showing the protein expression of (c) native Nox2 or (e) Nox2β in RAW264.7 cells following either vehicle or siRNA treatment and (d) and (f) their respective histograms showing densitometric analysis normalized to β-actin expression (n = 3). Superoxide production in the presence of PDB following either (g) vehicle or siRNA treatment (n = 6) or (h) scrambled siRNA or siRNA treatment. Statistical analyses were conducted using either a 1-way ANOVA with a Bonferroni multiple comparisons post-hoc test for PCR bands or a two-tail paired t-test for Western blotting and superoxide data. Statistical significance was taken when the P<0.05.

### siRNA

A small interfering RNA (siRNA) was designed to span the junction between exons 3 and 11 of the splice variant sequence. A scrambled siRNA made up of the same nucleotides, but in a random order, was used as a control. All siRNA were blasted against the mouse genome stored in the NCBI Genbank database to detect potential homology with non-Nox2 genes and transcripts. Only siRNA sequences with no significant homology were synthesized for testing. The siRNA sequences that were ultimately used are listed (5′ to 3′) below:

siRNA: 5′ AAGUAGAUCGCACUGGAAC[dT][dT] 3′
Scrambled: 5′ AGGAUAACGAUGUGCCAAC[dT][dT] 3′

Conditions for optimal transfection efficiency of siRNA in RAW264.7 (determined using a fluorescently (FAM) labelled siRNA sequence) were 100 nM siRNA and 3% of HiPerFect on cells of ∼40% confluency.

A flask of RAW264.7 cells was scraped and counted using the Countess® Automated Cell Counter (Invitrogen). 2.4×10^5^ cells were seeded into wells of a 12-well culture plate in duplicate per treatment group and incubated in growth media for 18 h. Following incubation, cells were rinsed of growth media with 2 ml of FCS-free media and then incubated for 6 h in 500 µl of either vehicle (3% HiPerFect made up in FCS-free media), siRNA (100 nM siRNA and 3% HiPerFect made up in FCS-free media) or scrambled control (100 nM scrambled siRNA and 3% HiPerFect made up in FCS-free media). Following the incubation period, the treatment solution was removed from the cells, which were then incubated for a further 18 h in 4 ml of normal growth medium. Following incubation, the cells were treated once more following the exact protocol outlined above, and then following a final 24 h incubation, one well from each treatment group was homogenised in Laemmli buffer (see: “Western blotting”) while the remaining well was homogenised for RNA extraction and RT-PCR reactions (see: “RNA extraction and Reverse Transcription”).

**Figure 9:**
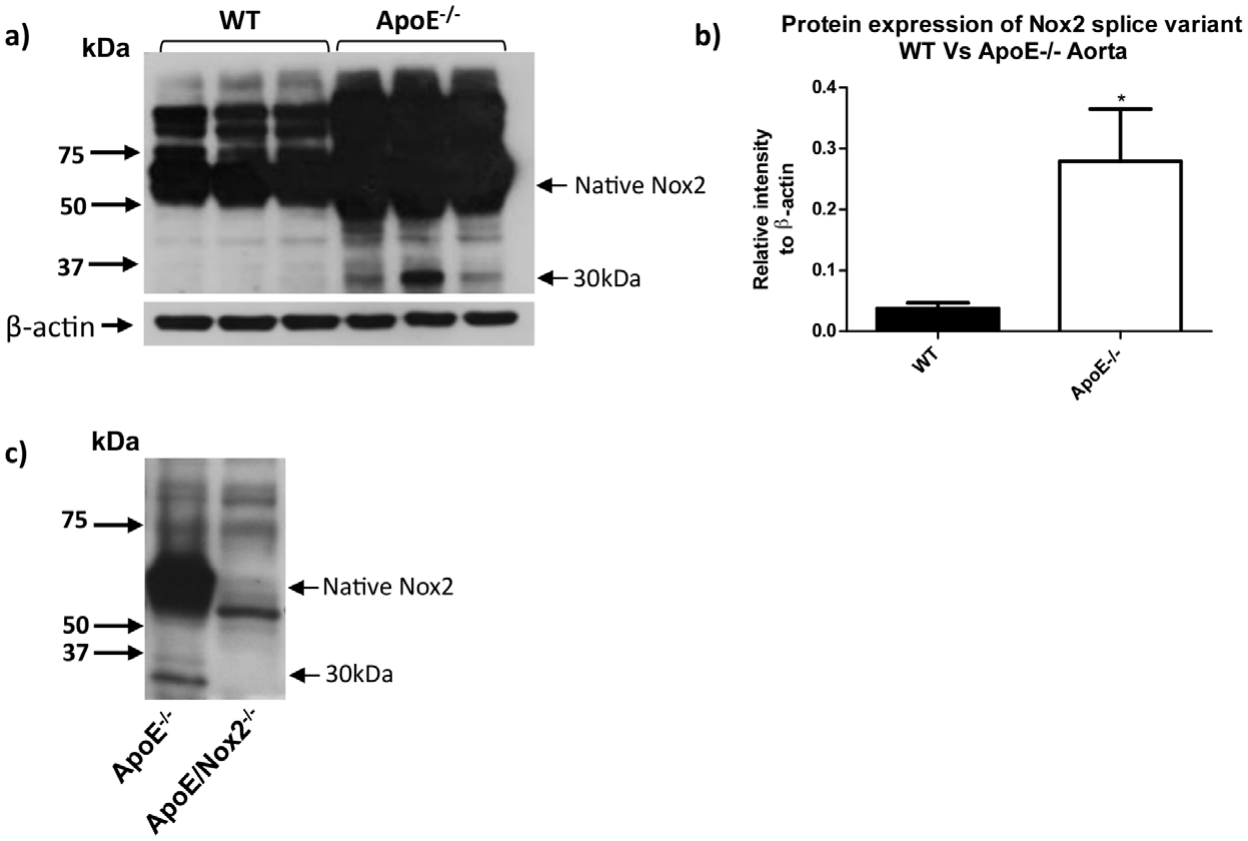
Expression of Nox2βin atherosclerotic arteries. (a) Western blots images and (b) corresponding densitometric analysis showing the expression of Nox2β and native Nox2 in mouse aorta from age-matched wild type mice and APOE^−/−^ mice on a high fat diet for 19–26 weeks. (C) Western blot image showing the lack of expression of Nox2β in aorta taken from the novel double APOE^−/−/^Nox2^−/y^ mouse.

### Western Blotting

Lungs and aorta were snap-frozen in liquid nitrogen and pulverised using a mortar and pestle. Following pulverisation, 0.4–0.5 ml of Laemmli buffer (5% glycerin, 2.5% mercaptoethanol, 1.5% sodium dodecyl sulphate (SDS), 50 mmol/L Tris-HCl, pH 8, and 0.05 mg/mL bromphenol blue) was added to each sample.

Tissue and cell homogenates were then sonicated (cycle 0.5, amplitude 80%) for 10 s using a Hielscher ultrasonic processor (UPSOH, Germany) and then heated on a heating block at 37°C for 10 min. The supernatant was removed by centrifugation at 15,000×g for 5 min at 4°C, and samples were stored at –20°C until analysis.

Protein concentrations of primary cells and tissue samples were determined using the RC DC™ protein kit according to the manufacturer’s specifications. Matched protein amounts of varying concentration (depending on the tissue type) were loaded onto 10% polyacrylamide gels. Samples were then resolved by SDS–polyacrylamide gel electrophoresis and transferred onto a polyvinylidene fluoride (PVDF) membrane. Following transfer, membranes were blocked in 5% non-fat dry milk (Bio-Rad blotting-grade blocker) for 1 h at room temperature and then incubated overnight at 4°C with a Nox2 antibody, (monoclonal; BD Transduction Laboratories; dilution 1∶1000). Membranes were then washed three times at 10 min intervals with Tris-buffered saline-tween (TBS-T) and then incubated for 1 h at room temperature with anti-mouse secondary antibody (Jackson ImmunoResearch; dilution 1∶10000) conjugated to horseradish peroxidase. Membranes were again washed in TBS-T (3×10 min for native Nox2 and β-actin or 5×15 min for Nox2 variant) and immunoreactive bands were visualized following 1–30 min exposure to enhanced chemiluminescence (ECL) reagent (Blok-CH, Millipore) or (GE Healthcare ECL-advance) on X-ray film (Super RX; Kodak). Immunoreactive bands were quantified using ChemiDoc XRS Imager and Quantity One software (Bio-Rad). Following visualization, membranes were stripped in 0.5 M NaOH for 20 min and incubated with a primary antibody against β-actin (monoclonal; Sigma, St Louis, MO, USA; dilution 1∶5000) to assess protein loading and for normalization of immunoreactive bands.

### ROS production

4×10^5^ RAW264.7 cells were seeded into wells of a 96-well Viewplate (Packard). ROS production was measured using L-012-enhanced chemiluminescence. Cells were washed (x2) of media with 37°C Krebs-HEPES buffer and then exposed to a Krebs-HEPES buffer containing L-012 (100 µmol/L) and the protein kinase C (PKC) activator phorbol dibutyrate (PDB; 10 µmol/L). Photon emission (relative light units (RLU)/s) was detected using the Chameleon™ luminescence detector (Hidex, model 425105, Finland). Photon emission was recorded from each well for 3 s every 90 s over a period of 1 h and then averaged and normalised per mg protein per well. Protein amounts in each well were determined using the RC DC™ protein kit (Bio-Rad Laboratories, Hercules, CA, USA).

### Data Analysis

All densitometry data are expressed as either a percentage of vehicle or normalized to β-actin density. Statistical comparisons were made using one-way ANOVA with Bonferroni multiple comparisons test or two-tailed paired t-tests. ROS data are expressed as mean ± SEM with statistical comparisons made using two-tailed paired t-tests. All statistical tests were performed using GraphPad Prism version 5.04 (GraphPad Software, San Diego CA, USA). P<0.05 was taken to be significant.

### Bioinformatics

Sequence alignment and analysis was performed using DNASIS MAX bioinformatics software from Hitachi Solutions America Ltd. LUSTAL. A hydrophobicity plot was generated by “The Molecular Toolkit: Colorado State University”. Molecular modelling was performed using the TMpred software from European Molecular Biology Network (EMBnet). Potential N-glycosylation sites were identified using the NetNGlyc 1.0 Server (Centre For Biological Sequence Analysis, Denmark). Molecular weight predictions were calculated using the Swiss Institute of bioinformatics portal ‘ExPASy’.

## Results

### Truncated Nox2 Protein

Western blotting for Nox2 was performed on homogenates prepared from several mouse tissues known to express Nox2, including aorta, spleen, lung, kidney, heart and brain (Figure 1a). In all samples, a 58 kDa band, which is compatible with the MW of full length Nox2 protein [23], was readily detectable. In lung, and to a lesser extent in spleen, an additional immunoreactive band, with an apparent MW of ∼30 kDa, was detected (Figure 1a). Neither this band nor the 58 kDa full length protein were detected in lung samples from Nox2^−/y^ mice (Figure 1b).

A common feature of lung and spleen is that both organs contain large populations of resident macrophages. Given that macrophages express high levels of native Nox2 [24], we probed protein samples prepared from these cells for the presence of the 30 kDa truncated protein. We found that alveolar macrophages from wild type mice expressed high levels of the truncated Nox2 protein, as did mouse peritoneal macrophages, and the immortalised mouse macrophage cell line RAW264.7 (Figure 2). By contrast, the ∼30 kDa band was not detected in macrophages isolated from Nox2^−/y^ mice (Figures 2b and c). The truncated Nox2 protein was absent in circulating monocytes of wild type mice, suggesting that its expression is switched on after these cells differentiate into macrophages (Figure 2c).

### Truncated Nox2-specific RT-PCR Products Identified in Macrophages

We next employed RT-PCR on polyadenylated mRNA from primary peritoneal macrophages from wild type mice to determine if the truncated Nox2 protein identified above was potentially the product of alternative mRNA splicing. PCR with forward and reverse primers targeted to regions within exons 3 and 13 of the Nox2 gene, respectively, yielded multiple bands including a prominent band of ∼1400 base pairs – which is equivalent to the predicted size of the native Nox2 DNA amplicon – as well as a doublet consisting of bands between 300 and 400 base pairs in size (Figure 3a). These two bands were excised from the gel (together) and the DNA eluted from them was re-amplified for sequencing using the same primer set. As expected, this second reaction produced the same pair of lower MW products, but at a higher yield than the initial reaction, presumably due to the lack of competition for PCR products by the native Nox2 template (Figure 3b).

Sequencing of the 1400 base pair band and subsequent alignment with the published sequence (Genebank Accession No. NM_007807.4) confirmed that it was a positive match to the full length Nox2 message (see sequence #1; Figure 4). Regarding the truncated doublet, DNA sequencing and alignment revealed that the higher MW band (∼390 base pairs) was also homologous with the published Nox2 mRNA sequence, except that it lacked a large section of nucleotides in the middle of the transcript, corresponding to exons 4 to 10 of the Nox2 gene (see sequence #2 Figure 4). Sequencing of the lower MW band (∼360 base pairs) indicated that it was not related to Nox2 and thus likely to be the result of non-specific cDNA amplification (see sequence #3 Figure 4). Using a similar PCR amplification and re-amplification strategy and subsequent sequencing, we confirmed that message for both native Nox2 and the truncated variant lacking exons 4 to 10 was also expressed in RAW264.7 cells (Figure 3c and 3d and see sequences #4 and #5 from Figure 4).

To further confirm expression of the truncated Nox2 transcript in RAW264.7 cells, and to obtain its complete ORF, PCRs were performed with primers designed to bind to either the 5′ or 3′ UTR of the Nox2 mRNA. These primers were used in combination with a reverse or forward primer, respectively, designed to span the predicted splice site (SS) resulting from deletion of exons 4 to 10 (Figure 5a). Reactions using the 5′ UTR forward primer and the SS reverse primer yielded a predominant PCR product of ∼300 base pairs (Figure 5b), which is the predicted size of the amplicon assuming that exons 1–3 were intact in the truncated splice variant, and indeed sequencing confirmed this to be the case (see sequence #6 Figure 4). Reactions containing the SS forward primer and a 3′ UTR reverse primer yielded a single 450 base pair product (Figure 5c). This is the size of the amplicon that would be expected if the splice variant contained exons 11 to 13 intact, and again this was confirmed by DNA sequencing (see sequence #7 Figure 4). It is noteworthy that when the SS forward primer was replaced with a primer designed to bind entirely within exon 3, the truncated transcript was still amplified, but in markedly lower amounts due to the competing reaction that resulted in amplification of the native Nox2 transcript (Figure 5c).

Alignment of the mouse and human Nox2 sequences revealed an extremely high degree of homology at the respective splice site junctions predicted to be formed by the deletion of exons 4–10 (Figure 5d). Therefore, to determine whether a similar splicing event may occur in human cells, RT-PCR was performed on human alveolar macrophages using the same SS reverse primer as above, in conjunction with a forward primer designed to bind to a region within exon 1 that was highly homologous between the mouse and human. This reaction generated a product of 266 base pairs, which is identical to the size of the predicted amplicon (Figure 5e). Moreover, a similar sized product was generated when the same PCR was performed on cDNA from RAW264.7 cells (Figure 5e).

### Characteristics of the Predicted Protein

The above sequencing data revealed that despite the magnitude of the nucleotide deletion, the splicing event that gives rise to the truncated Nox2 variant does not result in a shift of the ORF. The Nox2 splice variant is predicted to yield a protein that is homologous to native Nox2, but missing those amino acids encoded by exons 4 to 10 (Figure 6a). These amino acids are primarily responsible for making up the transmembrane spanning domains of the full length Nox2 protein. The unmodified predicted protein is estimated to be ∼ 24.7 kDa in size and a hydrophobicity plot of the predicted amino acid sequence showed two regions of significant hydrophobicity at the N-terminus, which are predicted to represent transmembrane domains, as well as a long hydrophilic C-terminal tail (Figure 6b). Topology prediction algorithms place this C-terminus within the cell cytosol (Figure 6c).

The theoretical protein translated from the splice variant was aligned with native Nox2 protein and examined for the retention of functional domains (Figure 7a). The variant sequence appears to retain a number of important functional domains including two putative p47phox and three NADPH binding sites. In addition, the variant contains a possible N-glycosylation motif on the putative extracellular loop that joins the two transmembrane domains, but it does not retain any of the four histidine residues reported to bind the heme-containing porphyrin rings that are thought to be required for the production of superoxide by native Nox2 oxidase. Figure 7b is a schematic of the predicted protein generated by the Nox2 splice variant.

### Small Interfering RNA-mediated Gene Knockdown Reveals a Functional Role for the Novel Nox2 Splice Variant

To determine whether the Nox2 splice variant identified above has a functional role in ROS production by macrophages, we selectively knocked down its expression with siRNA designed to bind across the splice site boundary between exons 3 and 11. Treatment with this siRNA, but not with a scrambled siRNA sequence, significantly reduced mRNA expression of the truncated variant (Figure 8a and b). Accordingly, expression of the 30 kDa Nox2 protein variant was also reduced, while expression of the full length (58 kDa) protein remained unchanged (Figures 8c to f). Finally, siRNA-mediated knockdown of the Nox2 splice variant was associated with a 50% reduction in PDB-stimulated ROS production in RAW264.7 cells compared to vehicle-treated cells (Figures 8g and h).

### Vascular Expression of the Truncated Nox2 Protein is Elevated during Atherosclerosis

Macrophages infiltrate blood vessels during atherogenesis and contribute to plaque formation via Nox2-dependent ROS production [11]. As reported previously [11], aortas from ApoE^−/−^ mice maintained on a high fat diet for between 14–21 weeks displayed a marked increase in expression of native Nox2 compared with age-matched wild type mice (Figure 9). Furthermore, the ∼30 kDa Nox2 variant was also readily detectable in aortas from ApoE^−/−^ mice, whereas it was absent in tissues from either wild type or Nox2^−/y^/ApoE^−/−^ double knockout mice (Figure 9).

## Discussion

This study has identified a novel splice variant of Nox2 that is expressed in mouse and human macrophages and is an important contributor to the NADPH oxidase activity of these cells. As only the second splice variant of Nox2 to be identified, we propose the name Nox2β.

Nox2β, which appears to be the product of the ‘exon skipping’ mode of alternative splicing, lacks the portion of mRNA encoded by exons 4 to 10 of the Nox2 gene. The variant is predicted to yield a protein product that is 216 amino acids long with a molecular weight (in its unmodified form) of 24.7 kDa. Using an anti-Nox2 antibody raised against an epitope contained within the C-terminal tail of native Nox2 (which is predicted to be retained in Nox2β), we observed a truncated immunoreactive band in macrophage samples and lung homogenates from wild type mice that was ∼30 kDa in size. This protein was absent in macrophage and lung samples from Nox2^−/y^ mice, and was down-regulated following treatment of cultured macrophages with siRNA designed to selectively target the truncated mRNA variant. We propose that the ∼5 kDa difference in actual and predicted size of the Nox2β protein product is likely due to the retention of a putative N-glycosylation site. Indeed, for native Nox2, which also contains only one likely N-glycosylation site, the addition of sugar side-chains results in ∼4 kDa increase in molecular weight from 54 kDa to 58 kDa [22].

Nox2β is distinct from Nox2S, the other Nox2 splice variant described. The ORF of Nox2S includes exons 1 to 3 of the Nox2 gene, as well as a previously unidentified exon (denoted IIIa) mapping 6.4 kB downstream of exon 3 and containing an in-frame stop codon. The predicted protein product of Nox2S is only 12.7 kDa in size. It contains two transmembrane domains and a C-terminal tail that is devoid of NADPH or FADH binding domains, but instead contains phosphorylation sites for protein kinase C. The distribution pattern of Nox2S appears to be similar to that of the variant described in this paper with both lung and spleen displaying the highest expression levels. Furthermore, Nox2S was expressed at very low levels in the monocytic cell line HL-60, but was induced when these cells were transformed into macrophages by treatment with dimethyl sulfoxide. However, the functional significance of Nox2S was not investigated.

The C-terminus of Nox2β retains two protein motifs that are reported to be involved in the binding of the NADPH oxidase organiser subunit, p47phox, to the full length Nox2 protein [25]. This raises the possibility that Nox2β may compete with native Nox2 for p47phox binding *in vivo* and thereby modulate NADPH oxidase activity. Therefore, we examined the impact of siRNA-mediated gene silencing of Nox2β on ROS production by RAW264.7 cells following stimulation with PDB. In resting cells, p47phox is present in the cytosol in an auto-inhibited conformation as part of a heterotrimeric complex with the NADPH oxidase activator subunits, p67phox and p40phox. Phorbol esters activate protein kinase C and thereby induce phosphorylation of p47phox, causing it to become freed from its auto-inhibited state and allowing it to interact with the Nox2 catalytic subunit to stimulate NADPH oxidase activity. As expected, stimulation of RAW264.7 cells with PDB caused a rapid and dramatic increase in ROS production. Gene knockdown of Nox2β reduced the magnitude of PDB-stimulated ROS production by ∼50%, suggesting that the truncated splice variant plays an important role in regulating ROS production by macrophages.

The finding that Nox2β facilitates ROS generation is surprising given that the variant lacks several protein regions that are known to bind prosthetic groups involved in the transfer of electrons from NADPH to molecular oxygen within native Nox2. These include four conserved histidine residues that would normally be contained within the transmembrane domains and which are postulated to bind two Fe-heme complexes, as well as a FADH binding site normally located at the base of the cytosolic C-terminal tail. A previous study investigating several naturally occurring splice variants of Nox4 revealed that one such variant - Nox4D - when overexpressed in a pulmonary epithelial cell line, generated H_2_O_2_ in levels that were comparable to those generated by native Nox4 [19]. This was in spite of the fact that Nox4D only retained one of the six transmembrane domains of native Nox4, lacked the four conserved histidine residues involved in anchoring the Fe-heme, and was also missing a significant portion of the cytosolic FADH binding domain [19]. It remains to be determined whether Nox2β, or indeed Nox4D, can directly transfer electrons from NADPH to molecular oxygen to generate ROS. Alternatively, it is possible that these splice variants represent novel regulatory proteins that are incorporated into their respective NADPH oxidase complexes to enhance the catalytic activity of the full length Nox proteins. Either way, the structural and functional similarities between Nox2β and Nox4D raise the possibility that the splicing events responsible for their generation may have been conserved during the evolution of the mammalian Nox family members, lending further support to the concept that these proteins play important functional roles in regulating NADPH oxidase activity.

The implications of a novel splice variant of Nox2 capable of promoting ROS in macrophages are potentially profound. Nox2 oxidase-derived ROS in macrophages play a crucial role in innate immunity and defence against invading microorganisms. Furthermore, excessive Nox2 oxidase activity is an important contributor to the oxidative stress and tissue pathologies of several chronic diseases such as hypertension, diabetes and atherosclerosis [26], [27]. Further to this latter point, it was interesting to note that expression of Nox2β was markedly elevated in the aortic wall of atherosclerotic ApoE^−/−^ mice. The expression of Nox2β in atherosclerotic aorta is most likely to be a consequence of the infiltration of macrophages; however, expression of the variant in other cells of the vascular wall such as endothelial cells cannot be ruled out. We have previously shown that deletion of the Nox2 gene in ApoE^−/−^ mice affords protection against endothelial dysfunction and atherosclerotic lesion development [11]. Deletion of the Nox2 gene in these animals would have resulted in a loss of both native Nox2 and Nox2β activity. Hence, the relative contributions of the two proteins to aortic superoxide production and oxidative stress, and ultimately to the development of atherosclerotic lesions, remains to be determined. To address this fully, future studies utilizing tools (e.g. siRNA) that selectively silence Nox2β or Nox2 in vivo, and multiple models of atherosclerosis (i.e. ApoE^−/−^ and LDLR^−/−^ mice), are required.

In conclusion, this study has identified a novel splice variant of Nox2– Nox2β - that is expressed in mouse and human macrophages and promotes NADPH oxidase-dependent ROS generation in these cells. It remains to be determined what role(s) Nox2β plays in physiological and pathophysiological conditions, however, given that a large amount of current knowledge about Nox2 is based on studies using gene knockout mice, which would lack both variants, it is conceivable that some of the previously defined roles of full length Nox2 may be attributable to the actions of Nox2β.
